# Food Insecurity and Dietary Quality in African American Patients with Gastrointestinal Cancers: An Exploratory Study

**DOI:** 10.3390/nu16183057

**Published:** 2024-09-11

**Authors:** Daaimah Dratsky, Erin McGillivray, Juhi Mittal, Elizabeth A. Handorf, Giuliana Berardi, Igor Astsaturov, Michael J. Hall, Ming-Chin Yeh, Rishi Jain, Carolyn Y. Fang

**Affiliations:** 1 Department of Nutrition and Public Health, Hunter College, New York, NY 10035, USA; daaimah.dratsky@hunter.cuny.edu (D.D.); myeh@hunter.cuny.edu (M.-C.Y.); 2 Department of Internal Medicine, University of Southern California, Los Angeles, CA 90089, USA; 3 Department of Hematology/Oncology, Temple University Hospital, Philadelphia, PA 19140, USA; juhi.mittal@tuhs.temple.edu (J.M.); giuliana.berardi@tuhs.temple.edu (G.B.); 4 Department of Biostatistics and Epidemiology, Rutgers University, New Brunswick, NJ 08901, USA; ehandorf@cinj.rutgers.edu; 5 Department of Hematology/Oncology, Fox Chase Cancer Center, Philadelphia, PA 19111, USA; igor.astsaturov@fccc.edu (I.A.); michael.hall@fccc.edu (M.J.H.);; 6 Cancer Prevention and Control Program, Fox Chase Cancer Center, Philadelphia, PA 19111, USA; 7 Department of Clinical Genetics, Fox Chase Cancer Center, Philadelphia, PA 19111, USA

**Keywords:** food insecurity, diet quality, health disparities, African Americans, gastrointestinal cancers

## Abstract

African American (AA) individuals experience food insecurity at twice the rate of the general population. However, few patients are screened for these measures in the oncology setting. The primary aim of this study was to evaluate associations between food insecurity and dietary quality in AA patients with gastrointestinal (GI) malignancies. The secondary aim was to evaluate differences in dietary quality and the level of food insecurity between the participants at Temple University Hospital (TUH) vs. Fox Chase Cancer Center (FCCC). A single-arm, cross-sectional study was conducted, in which 40 AA patients with GI malignancies were recruited at FCCC and TUH between February 2021 and July 2021. Participants completed the US Adult Food Security Survey Module to assess the level of food security (food secure vs. food insecure). An electronic food frequency questionnaire (VioScreen^TM^) was administered to obtain usual dietary intake. Diet quality was calculated using the Healthy Eating Index 2015 (HEI-2015). Dietary quality and food insecurity were summarized using standard statistical measures. Overall, 6 of the 40 participants (15%) reported food insecurity, and the mean HEI-2015 score was 64.2. No association was observed between dietary quality and food insecurity (*p* = 0.29). However, we noted that dietary quality was significantly lower among patients presenting at TUH (mean HEI-2015 = 57.8) compared to patients at FCCC (mean HEI-2015 = 73.5) (*p* < 0.01). Food insecurity scores were also significantly higher in the TUH population vs. the FCCC population (*p* < 0.01).

## 1. Introduction

Food insecurity is defined by the US Department of Agriculture (USDA) as “a household level economic and social condition of limited or uncertain access to adequate food” [1]. In adults in the general population, food insecurity is associated with obesity and poor dietary quality, including a decreased consumption of fruits, vegetables, and dairy products [2,3]. Due to a variety of factors including lower socioeconomic status, African American (AA) individuals have at least twice the rate of food insecurity in comparison to the national average [4,5]. Furthermore, the COVID-19 pandemic has exacerbated this existing disparity, with an 80% increase in AA households reporting as food insecure after March 2020 compared to a 60% increase for the general population [6]. It is well documented that neighborhood conditions in AA communities may further complicate matters, as physical access to grocery stores and full-service supermarkets is often limited [7]. As previous research suggests, this limited access to grocery stores can lead to a higher consumption of easily accessible, less nutrient-dense foods and a lower consumption of fruits and vegetables, which ultimately results in poor overall dietary quality [8,9,10,11].

Food insecurity also occurs in cancer populations. A 2020 survey of cancer survivors identified from the New Mexico Tumor Registry reported that 10% of survivors who were food secure prior to their diagnosis became newly food insecure after their diagnosis. These newly food insecure individuals were more likely to delay or go without necessary prescription medications and cancer treatments [12]. Furthermore, cancer patients who were diagnosed with gastrointestinal (GI) cancers reported more food insecurity than individuals who were diagnosed with non-GI cancers [13].

While there are limited data regarding the impact of food insecurity on cancer outcomes, conditions associated with food insecurity (such as obesity, malnutrition, or poor dietary quality) have been linked to inferior cancer outcomes, including reduced survival [13,14,15]. During treatment, the identification and management of malnutrition and poor dietary habits are of the utmost importance in patients with GI malignancies who suffer from particularly high rates of malnutrition and sarcopenia due to a combination of tumor-, host-, and treatment-related factors that can cause GI disturbances and inadequate nutritional intake [15,16,17,18,19]. Yet, despite evidence that poor diet quality is associated with worse clinical outcomes, the use of food frequency questionnaires (FFQs) to derive diet quality indices has been limited in healthcare settings [20]. This is due, in part, to concerns about the length of many FFQs and the respondent’s ability to accurately recall portion sizes [20]. Recent developments incorporating technology-enabled tools, however, may address some of these limitations. For example, VioCare’s VioScreen™, a 20-minute, validated, computer-based, graphical FFQ, has been successfully used to obtain data on foods consumed, eating behaviors, portion sizes, and macronutrient/micronutrient intake in a variety of non-cancer populations [21].

Thus, the goal of this study was to assess whether food insecurity is associated with poorer dietary quality among AA patients with gastrointestinal cancers recruited from two cancer clinics [Fox Chase Cancer Center (FCCC) and Temple University Hospital (TUH)], and whether differences in dietary quality and food insecurity exist across sites.

## 2. Materials and Methods

### 2.1. Study Design

A single-arm cross-sectional study was conducted with 40 AA patients with GI malignancies presenting at FCCC and TUH from February 2021 to July 2021. Fox Chase Cancer Center (FCCC), an NCI-designated comprehensive cancer center, and Temple University Hospital (TUH), an academic safety-net hospital, are both part of the Temple University Health System and provide oncology care to the greater Philadelphia region. The study was approved by the Fox Chase Cancer Center Institutional Review Board (FCCC IRB #20-8017).

### 2.2. Study Participants and Recruitment

Patients with GI malignancies who presented at FCCC or TUH GI medical oncology clinics were screened for eligibility and recruited for participation in this study. Inclusion criteria included adults (≥18 years of age) self-reporting AA race, with a diagnosis of gastrointestinal cancer (including esophageal, gastroesophageal junction, gastric, small intestine, colorectal, anal, hepatocellular, pancreatic, and biliary tract). There was no eligibility restriction based on cancer stage or prior treatments received. Exclusion criteria included patients who were not cognitively able to consent or had physical or mental limitations that would prevent full participation in the study (e.g., dementia). Additionally, patients with chronic malabsorption syndromes, clinically significant bowel obstructions, or those that required a low-residue or low-fiber diet were excluded from the study.

Eligible patients who were interested in participating reviewed the consent form with the study investigator or other study staff and were allowed time for questions and a final consideration of study participation. All study participants were provided with a copy of the signed consent form. A convenience sample of 40 participants was enrolled across both sites.

### 2.3. Study Procedure

Data collection occurred at a single time point during the participant’s routine outpatient appointment, with a medical oncologist in the FCCC or TUH gastrointestinal oncology clinics. Participants were interviewed by the research staff to confirm their personal information (race, sex, marital status, education, and insurance type) and these answers were recorded on a Demographic Data Sheet. The research assistant (RA) administered the food security assessment (USDA Adult Food Security Survey Module) verbally to each patient. Next, the patient completed the computerized food frequency questionnaire (VioScreen™) on an iPad (Apple, Cupertino, CA, USA). After completion, the VioScreen™ FFQ used the data provided to offer personalized recommendations on specific ways one can improve their dietary quality score. While an improvement in dietary quality was not the primary aim of the study, the research team believed that it was important for the RA to review the personalized recommendations with the patient given the known lack of nutritional services available at cancer centers in the US [19].

### 2.4. Assessments and Measures

Demographics (including age, race, sex, insurance type, marital status, and education level) were assessed via a self-report questionnaire and medical chart review at baseline. Cancer characteristics (e.g., diagnosis, stage) were obtained from medical chart review.

The USDA developed a validated assessment of food insecurity known as the US Adult Food Security Survey Module, designed to evaluate the financial ability of a household to support food needs over a one-year period [22]. This 10-item questionnaire has been used in low-income and ethnic/racial minority groups [22] and includes questions regarding household and individual food-related challenges (e.g., running out of money to buy food, inability to afford to eat well-balanced meals, needing to decrease the size of meals due to inadequate money, or skipping meals). A response of “yes”, “often”, “sometimes”, “almost every month”, and “some months but not every month” was coded as ‘1’, whereas a response of “no” was coded as ‘0’ [22]. Scores were summed across all 10 items, with the total score ranging from 0 to 10. Per the scale instructions, a raw score of 0 was categorized as high food security, a raw score of 1–2 as marginal food security, a raw score of 3–5 as low food security, and a raw score of 6–10 as very low food security. In this study, participants with a raw score of 0–2 were coded as food secure and those with a raw score of 3–10 as food insecure.

VioScreen™ FFQ (http://www.viocare.com/vioscreen, accessed on 25 September 2019) software (version 2.98.0.671) was used to assess dietary quality [21]. VioScreen™ is a web-based dietary analysis that uses a graphical FFQ method of approximately 1200 food images and branched decision tree logic for data collection. The Vioscreen^TM^ FFQ collects information on the previous 90 days for about 155 food and beverage items under the following categories: Cereals and Breads; Cereals and Breads; Eggs and Meats; Mixed Chicken and Fish; Asian, Mexican and Soy Foods; Soups; Cheese and Dairy Products; Cheese and Dairy Vegetables; Garden Vegetables; Potatoes, Beans and Rice; Oil/Fat Used in Cooking; Sauces and Seasonings; Fruits; Sweets; Chips, Crackers and Snacks; Meal Replacement Drinks, Sports and Granola Bars; Milk, Coffee and Tea; Soft Drinks, Water and Juice; Alcoholic Beverages; and Supplements. Up to six graphical portion sizes were provided for all food and beverage items [23]. The participant data captured in the Vioscreen^TM^ FFQ were automatically compiled into a thorough report that outlines their diet patterns and provides a Healthy Eating Index 2015 (HEI-2015) score for the interpretation of dietary quality. The HEI-2015 score is a measure of dietary quality that assesses conformance with the 2015–2020 Dietary Guidelines for Americans [24] and is composed of 13 specific components: total fruits, whole fruits, total vegetables, greens and beans, whole grains, dairy, total protein foods, seafood and plant proteins, fatty acids, refined grains, sodium, added sugars, and saturated fats [25]. The two groups of the 13 components are adequacy and moderation components. Adequacy components represent the food and nutrient groups in which higher intake is encouraged and higher scores reflect higher intakes [25]. Moderation components represent food and nutrient groups that should be limited, and higher scores reflect lower intakes, which are desirable. Each of the 13 components is assigned a maximum score of 5 or 10 points and the total summed score (i.e., the HEI-2015 score) has a maximum value of 100 [25]. To distinguish the difference between the composite HEI-2015 score and the individual component scores, the HEI-2015 composite score measures overall diet quality while the component scores show the pattern in diet quality [25] HEI-2015 scores are interpreted using a graded approach:

- Overall scores of 90 to 100, or component scores that are 90% to 100% of maximum score: A;
- Overall scores of 80 to 89, or component scores that are 80% to 89% of maximum score: B;
- Overall scores of 70 to 79, or component scores that are 70% to 79% of maximum score: C;
- Overall scores of 60 to 69, or component scores that are 60% to 69% of maximum score: D; and
- Overall scores of 0 to 59, or component scores that are 0% to 59% of maximum score: F [25].

### 2.5. Statistical Analysis

The primary aim of this study was to evaluate the association between dietary quality and food insecurity. The secondary aim of this study was to evaluate whether diet quality differed between participants at TUH and FCCC. The threshold for statistical significance was set at 0.05 (two sided). R Statistical Software (version 3.6) was used for the data analysis in this study. Dietary quality and food insecurity were summarized using standard statistical measures (means, standard deviations, and significance testing). Differences between groups were tested using the Wilcoxon rank test or the Fischer’s test.

## 3. Results

Forty participants were recruited and enrolled in the study. All 40 participants completed the study assessments, giving a completion rate of 100%.

Table 1 presents the demographic characteristics of the study sample and the comparison across sites. In total, fifty-eight percent (*n* = 23) of participants were female (*n* = 13; 54% F at TUH and *n* = 10; 62.5% F at FCCC). The mean age was 63 (+/−13) years. The majority (21; 53%) were diagnosed with colorectal cancer, followed by pancreatic cancer (5; 12.5%). Both late and early cancer stages were represented in this study, with 35% (14) having early-stage and 65% (26) having late-stage (metastatic) disease. The majority of participants (31; 77.5%) were in active treatment, 5% (2) in post-operative, and 17.5% (7) in surveillance. Further, 5% percent of the participants (2) were classified as underweight (BMI < 18.5), 25% (10) normal weight (BMI: 18.5–24.9), 32.5% (13) overweight (BMI: 25–29.9) and 37.5% (15) obese class I to III (BMI ≥ 30). Most participants had Medicare coverage as their primary form of insurance (20; 50%), but participants at FCCC were more likely to have private insurance coverage (10; 62.5% compared to 4; 16.7% at TUH, *p* = 0.05). Education levels differed significantly between the participants at TUH vs. FCCC (*p* = 0.02), with 56.2% (9) of participants at FCCC completing some college or more compared with only 12.5% (3) of TUH participants. Most TUH participants did not complete high school (12; 50%) compared with 12.5% (2) of FCCC participants. At both sites, the most common marital statuses were Single (17; 42.5%) and Married/Living with Partner (14; 35%).

Across both sites, six participants (15%) had US Adult Food Security Survey Module total scores of ≥3 (which indicates food insecurity). Five participants (12.5%) at TUH had total scores of ≥3 compared to one participant at FCCC (2.5%) (*p* = 0.04).

**Figure 1:**
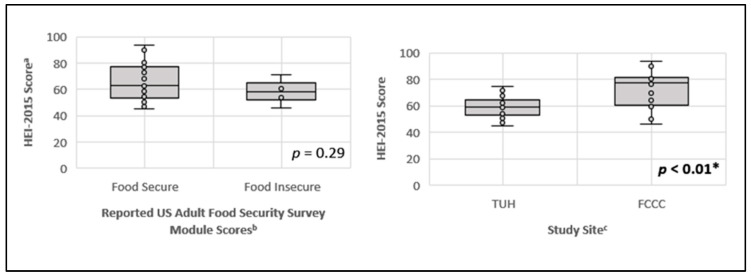
Associations between diet quality and US Adult Food Security Survey Module Scores, and diet quality and study site, for 40 African American patients with gastrointestinal cancers. ^a^ Healthy Eating Index-2015, a measure of diet quality. ^b^ Food security scores measured by the US Adult Food Security Survey Module (10 items survey, range 0–10). Raw scores of 0–2 indicate the participant is food secure and raw scores 3–10 indicate food insecure. In this study, participants with scores of 3 or greater were categorized as food insecure/having food insecurity [24]. ^c^ TUH = Temple University Hospital and FCCC = Fox Chase Cancer Center. * Statistically significant difference between study sites.

Figure 1 shows the evaluation of reported US Adult Food Security Modules scores and study site (TUH and FCCC) and their associations with the reported HEI-2015 scores. No significant relationship was found between the level of food security (food secure vs. food insecure) and the HEI-2015 scores (*p* = 0.29). Specifically, although mean dietary quality was higher among participants categorized as food secure (*n* = 34) (M = 65.5, SD = 12.9) than participants with food insecurity (*n* = 6) (M = 58.5, SD = 8.6), this difference did not reach statistical significance. We also examined whether potential sociodemographic factors were associated with HEI-2015 scores. There was a negative association between age and diet quality (*r =* −0.31, *p* = 0.05), with older participants reporting lower diet quality. Marital status and insurance status were not associated with diet quality, but participants with a high school education or less had a lower diet quality (M = 61.2, SD = 11.0) compared with participants who had attended some college or beyond (M = 72.1, SD = 13.0), *p* < 0.01.

Further, there was a significant relationship between study site and HEI-2015 scores (*p* < 0.01). FCCC participants (*n* = 16) had a significantly higher diet quality (M = 73.5, SD = 12.4) compared to TUH participants (*n* = 24) (M = 57.8, SD = 8.0). This finding may be attributed, in part, to the sociodemographic differences (e.g., in education level) observed across sites.

To further explore the differences observed across study sites, we examined the HEI-2015 component scores. As shown in Table 2, TUH participants had significantly lower mean HEI-2015 component scores compared to FCCC participants in several categories, including Whole Grains, Fatty Acids, and Added Sugars (2.5, 4.5, and 6.4, at TUH vs. 5.2, 6.8, and 7.7 at FCCC, *p* < 0.01, *p* = 0.01, *p* = 0.04 respectively). Neither TUH nor FCCC participants met the guidelines for Whole Grains, Dairy, Sodium and Fatty Acids, as indicated by the lower scores out of 10. The mean HEI-2015 score for all participants was 64.2. The mean HEI-2015 score was 57.8 at TUH and 73.5 at FCCC. Participants at FCCC had a mean total score between 70 and 79, representing Grade C, and participants at TUH had a mean total score below 60, or Grade F.

## 4. Discussion

This study aimed to assess the frequency of food insecurity and its association with dietary quality among a sample of AA patients with GI malignancies. The rate of 15% for food insecurity in this study is consistent with the rates found in studies of cancer populations of all races (4–26%) [26]. However, this rate was not congruent with other studies that reported higher rates of 20–33% for food insecurity among African American cancer patients/survivors [12,27]. One possible reason for the lower rates of food insecurity could be due to specific policy measures enacted during the time in which this study was completed. In March 2020, the USDA expanded SNAP benefits in response to the COVID-19 pandemic, increasing benefits by 15% [28]. Additionally, in April 2021 while the study was ongoing, SNAP benefits were increased for all households by at least USD 95 to ensure low-income families could override maximum benefits [28]. This temporary expansion may have artificially depressed the rates of food insecurity in this study. The mean HEI-2015 total scores for the participants in our study (64.2) were higher compared to the mean total scores reported for cancer survivors in an evaluation study of 2016 NHANES data (total population survivor scores of 55.6, and 55.5 for AA survivors specifically) [29].

While no significant associations were observed between food insecurity and dietary quality, this study did reveal key differences in food insecurity and dietary quality between participants at TUH vs. FCCC. These differences could be explained in part by notable socioeconomic differences in patient populations across sites. TUH is a safety net hospital located in North Philadelphia, where the median household income is USD 16,700, 25% of residents have less than a high school education, a third receive SNAP benefits, and 40.6% of families live below the poverty line [30]. In comparison, Fox Chase Cancer Center is in the Philadelphia suburbs, where the median household income is USD 56,800, only 10% are without a high school education, 10% receive SNAP benefits, and 15.7% of families live below the poverty line [31]. Our results are in line with other studies, which have reported that lower household income and limited education are associated with greater food insecurity [4,32,33]. In addition, FCCC receives patient referrals from a broader region, has a greater volume of oncology patients, and attracts patients seeking access to specific clinical trials.

The present findings highlight the value and importance of integrating food security and dietary quality assessments into the oncology setting, as this may facilitate early intervention when warranted. A recently published pilot study noted that providing nutritious meal kits or no-prep meals resulted in significant improvements in perceived dietary quality and food security and was well-received in an underserved population [34]. Local and regional organizations may also offer valuable resources for improving diet quality. For example, the Metropolitan Area Neighborhood Nutrition Alliance (MANNA) Institute in Philadelphia is a community-based organization with a long history of providing medically tailored meals to individuals who are experiencing a serious illness, including cancer. Their comprehensive program includes dietary counseling by a registered dietitian combined with the delivery of 21 frozen medically tailored meals per week [35]. Importantly, studies noted a significant decrease in healthcare costs following the initiation of MANNA services when compared to a group of individuals who did not receive these services [36]. Thus, future studies could explore whether providing personalized nutrition recommendations along with nutritious meal kits or home-delivered meal services is effective in enhancing diet quality and health outcomes among patients with cancer.

Several study limitations should be noted. First, a key limitation is the small sample size of 40 participants, leading to larger variability among individuals in both the US Adult Food Security Survey Module scores and HEI-2015 scores. Further studies with larger sample sizes and across multiple populations are needed to verify the prevalence of food insecurity and its relationship with diet quality in the oncology setting. Second, to reduce participant burden, the study measures were limited in their focus. In addition to the USDA survey, future studies should collect information from participants pertaining to their household income, meal assistance program participation, and availability of full-service supermarkets. These added measures can provide a more comprehensive picture of participants’ food security status and their ease of access to nutritious foods. However, despite these limitations, this study had several strengths, including the use of validated measures to assess food security status (USDA) and diet quality (HEI-2015) in a sample of AA patients with gastrointestinal cancers, an understudied population at-risk for poor outcomes.

## 5. Conclusions

No significant associations were observed between the levels of food security (food secure vs. food insecure) and diet quality in this sample. However, adherence to the Dietary Guidelines was fair to poor, as reflected in the graded HEI-2015 scores of C or below. Further research with a larger sample is warranted to identify innovative approaches to improve diet quality in African American patients with cancer.

## Figures and Tables

**Table 1:** Demographics and composite food security scores of 40 African American patients with gastrointestinal cancers at TUH and FCCC participating in an exploratory cross-sectional analysis on the links between food insecurity and diet quality.

Variable	Total (*n* = 40)	Temple University Hospital (*n* = 24)	Fox Chase Cancer Center (*n* = 16)	*p* Value
Age (years), mean (SD ^a^)	63 (13)	66 (10)	58 (15)	0.05
BMI (kg/m^2^), *n* (%)				0.66
Underweight (BMI < 18.5)	2 (5%)	4(16.7%)	2 (12.5%)	
Normal (18.5–24.9)	10 (25%)	4 (16.7%)	3 (18.7%)	
Overweight (25–29.9)	13 (32.5%)	6 (25%)	7 (43.8%)	
Obese, Class I (30–34.9)	7 (17.5%)	5 (20.8%)	3 (18.7%)	
Obese, Class II (35–39.9)	4 (10%)	2 (8.3%)	1 (6.3%)	
Obese, Class III (≥40)	4 (10%)	3 (12.5%)	0 (0%)	
Gender, *n* (%)				0.74
Male	17 (42%)	11(46%)	6 (37.5%)	
Female	23 (58%)	13 (54%)	10 (62.5%)	
Cancer Type, *n* (%)				0.51
Colorectal	21 (53%)	12 (50%)	9 (56.3%)	
Pancreatic	5 (12.5%)	3 (12.5%)	2 (12.5%)	
Liver	4 (10%)	4 (16.7%)	0 (0%)	
Gastric	3 (7.5%)	2 (8.3%)	1 (6.3%)	
Gallbladder/Biliary Tract	3 (7.5%)	1 (4.2%)	2 (12.5 %)	
Esophageal	2 (5%)	1 (4.2%)	1 (6.3%)	
Small Bowel	1 (2.5%)	0 (0%)	1 (6.3%)	
Anal	1(2.5%)	1 (4.2%)	0 (0%)	
Treatment Phase, *n* (%)				0.02
Active Treatment	31 (77.5%)	15 (62.5%)	16 (100%)	
Post-operative	2 (5%)	2 (8.3%)	0 (0%)	
Surveillance	7 (17.5%)	7 (29.2%)	0 (0%)	
Stage, *n* (%)				0.75
Early	14 (35%)	9 (37.5%)	5 (31%)	
Late	26 (65%)	15 (62.5%)	11 (69%)	
Insurance Type, *n* (%) ^b^				0.03
Private	14 (35%)	4 (16.7%)	10 (62.5%)	
Medicare	20 (50%)	14 (58.3%)	6 (37.5%)	
Medicaid	12 (30%)	9 (37.5%)	3 (18.8%)	
Education, *n* (%)				0.02
Some High School	14 (35%)	12 (50%)	2 (12.5%)	
High School Diploma/GED	14 (35%)	9 (37.5%)	5 (31.3%)	
Some College or More	12 (30%)	3 (12.5%)	9 (56.2%)	
Marital Status, *n* (%)				0.22
Single	17 (42.5%)	8 (33.3%)	9 (56.2%)	
Married/Living with Partner	14 (35%)	8 (33.3%)	6 (37.5%)	
Divorced	4 (10%)	4 (16.7%)	0 (0%)	
Widowed	5 (12.5%)	4 (16.7%)	1 (6.3%)	
Food Security Scores ≥ 3, total (%) ^c^	6 (15%)	5 (20.8%)	1 (6.3%)	0.04
HEI-2015 ^d^ Score, mean (SD)	64.1 (12.6)	57.8 (8.2)	73.5 (14.3)	<0.01

Note: ^a^ SD = Standard Deviation; ^b^ Some participants have more than one form of insurance, so percentage totals are >100%; ^c^ Food security scores measured by the US Adult Food Security Survey Module (10 item survey, range 0–10), raw scores of 0–2 indicate the participant is food secure and raw scores 3–10 indicate food insecure. In this study, participants with scores of 3 or greater were categorized as food insecure [22]; ^d^ The HEI-2015 or Healthy Eating Index-2015 total score consists of 13 individual components. Each of the 13 components is assigned a maximum score of 5 or 10 points, and the total summed score of these 13 components has a maximum value of 100 [25].

**Table 2:** HEI-2015 Total and component scores derived from Vioscreen FFQ for 40 African American patients with gastrointestinal cancers at TUH and FCCC.

HEI-2015 ^a^ Components	Mean Component Scores-Both Sites (*n* = 40)	TUH ^c^ Mean Component Scores (*n* = 24)	FCCC ^d^ Mean Component Scores (*n* = 16)	*p* Value
Adequacy (increased consumption is reflected by higher scores)				
Total Fruits (5) ^b^	4.5	4.2	4.9	0.44
Whole Fruits (5)	4.6	4.3	5.0	0.25
Total Vegetables (5)	3.8	3.7	4.1	0.26
Greens and Beans (5)	3.9	3.6	4.4	0.07
Whole Grains 10)	3.5	2.5	5.2	<0.01
Dairy (10)	5.6	5.3	6.1	0.99
Total Protein Foods (5)	4.1	3.9	4.4	0.75
Seafood and Plant Proteins (5)	3.7	3.3	4.4	0.21
Fatty Acids (10)	5.4	4.5	6.8	0.01
Moderation (reduced consumption is reflected by higher scores)				
Refined Grains (10)	7.8	7.1	8.8	0.16
Sodium (10)	4.2	4.1	4.4	0.76
Saturated Fat (10)	6.0	5.1	7.3	0.07
Added Sugars (10)	6.9	6.4	7.7	0.04
Total Score (100)	64.2	57.8	73.5	0.01

Note: ^a^ Healthy Eating Index-2015. ^b^ The HEI-2015 total score consists of 13 individual components. Each of the 13 components is assigned a maximum score of 5 or 10 points as noted by component (5) or component (10). The total summed score of these 13 components has a maximum value of 100 [25]. ^c^ TUH = Temple University Hospital. ^d^ FCCC = Fox Chase Cancer Center.

## Data Availability

Data from the present study are not publicly available due to their containing protected health information that could compromise the privacy of research participants, but are available from the corresponding author on reasonable request.
